# A model to explain road traffic data collection and registry in Iran: a grounded theory

**DOI:** 10.5249/jivr.vo113i2.1406

**Published:** 2021-07

**Authors:** Sakineh Sharifian, Reza Khani Jazani, Davoud Khorasani-Zavareh, Homayoun Sadeghi-Bazargani, Mohammad Hossein Vaziri, Reza Mohammadi

**Affiliations:** ^*a*^ Department of Nursing Management, School of Nursing and Midwifery, Iran University of Medical Sciences, Tehran, Iran.; ^*b*^ Department of Health in Emergencies and Disasters, School of Public Health and Safety, Shahid Beheshti University of Medical Sciences, Tehran, Iran.; ^*c*^ Safety Promotion and Injury Prevention Research Center, Shahid Beheshti University of Medical Sciences, Tehran, Iran.; ^*d*^ Traffic Injury Research Center, Statistics and Epidemiology Department, Tabriz University of Medical Sciences, Tabriz, Iran.; ^*e*^ Workplace Health Promotion Research Center (WHPRC), Shahid Beheshti University of Medical Sciences, Tehran, Iran.; ^*f*^ Department of Neurobiology, Care Sciences and Society (NVS), H1, Division of Family Medicine and Primary Care, Alfred Nobels Allé 23 141 83 Huddinge, Sweden.

**Keywords:** Registry system, Road traffic injury, Grounded theory, Qualitative study

## Abstract

**Background:**

Sufficient data should be gathered and analyzed to increase awareness and attention of the community and policymakers in the field of road traffic injury (RTI) prevention. While various organizations and stakeholders are involved in road traffic crashes, there is no clear lead agency for data collection system in RTIs. Exploring stakeholders' perspective is one of the key sources for understanding this system. The purpose of this study is to identify the process of RTI data collection system based on stakeholders’ experience.

**Methods:**

This qualitative study was conducted employing grounded theory approach since September 2017 to December 2018 in Iran. Participants in this study were the authorities of the Emergency organizations, police, Ministry of Health and Medical Education, faculty members, as well as executive staff and road users who were involved in collecting and recording data (n=15). Data collection was carried out through face-to-face interviews using purposeful and theoretical sampling. Data analysis was performed based on Strauss and Corbin 2008.

**Results:**

The core category was identified as “separated registration” explaining the process of collecting and recording road traffic injury data. Other variables obtained using the Strauss and Corbin Paradigm model were categorized as context, casual, intervening, strategies, and outcomes factors. The findings were classified into five groups including lack of trust in road safety promotion, process factors, management and organizational factors, failure of quality assurance, and administrative and organizational culture.

**Conclusions:**

The most important theory is “separated registration” and non-systematic registry system of road traffic injury data which is shown in a conceptual model. The findings of this study will help policymakers for better understanding the collecting and recording of RTI information.

## Introduction

One of the basic elements of sustainable development in high- and middle-income countries is the transportation system of each country. Insufficient attention to the promotion and improvement of the transportation and traffic system can impose many economic and social losses on countries and endanger the lives of many active people in society.^1^ Road Traffic Injuries (RTIs) account for almost 1.3 million deaths per year around the world and the 9th cause of burden of disease. Regarding to the World Health Organization (WHO) report, in Iran they are the second cause of death with almost 11% of mortality annually.^2^

Many of the injuries, including traffic injuries, are costly, but can be predictable. Therefore, valid and sufficient data should be collected and analyzed to increase awareness and attention of the community and policy makers of the country to the traffic field.^3^ Traffic injury data play an important role in identifying trends, groups and places of high risk, identifying risk factors for road crash and injuries, designing interventions and effective strategies, facilitating decision making for policy makers in this area, monitoring and evaluating programs in line with the goals.^4,5^

The data are gathered and recorded in many countries but they should be up to date and valid to be applied in roads safety improvement system. According to the World Road Safety Report, out of 178 countries, only 22% of countries report mortality and traffic crash injuries, economic outcomes and some information like vehicle speed, seat belts, alcohol and drug abuse.^6^ Lack of valid data regarding crashes and injuries is recognized as one of the major barriers to the advancement of programs and injury prevention interventions in middle- and lower-income countries.^7,8^ The prevention or reduction of traffic crash mortality is possible through the cooperation and participation of various departments and stakeholders in this field, including the transportation, health, police and training sectors, in relevant programs and facilities.^9^

Currently, due to various organizations and stakeholders in the field of traffic, there is no clear status of the system for collecting and recording traffic injury data in Iran.^10^

Studies have shown that data on traffic crash is not of required quality, and the information collected for the purpose of preventing and reducing traffic crash is not sufficient.^11-15^ So far, no study has been conducted to track the process of data collection and recording of traffic crashes, as well as the theory that describes the nature of the phenomenon of recording injury data in Iran. The discovery of stakeholders in collecting and recording traffic crash data is one of the most important sources for understanding this process. In this study, we intend to analyze this phenomenon from the perspective of the stakeholders in collecting and recording traffic crash data, so that by recognizing this process, measures and intervention to improve crash data registering can be considered as one of the main components of designing and implementation of the Road Traffic Injury (RTI) surveillance system.

## Methods 

**Type of study**

A qualitative study was designed and implemented employing grounded theory approach to conduct this research. The method of grounded theory is an effective way to understand and a comprehensive approach on the phenomena of interest.^16^ Moreover, considering the nature of interdisciplinary studies and the process of the phenomena, the most appropriate method for conducting these studies is the grounded theory.

In this method, the researchers attempt to explain the key processes and structures that are extracted from the experimental data and using the views and experiences of the beneficiaries in collecting and recording injury data and road traffic crash mortality, tries to answer the question that how the process of collecting and recording information is defined? And what are the casual factors? What are the strategies to deal with it? And what are the outcomes? This study was conducted through a deep face-to-face interview with stakeholders on the collection and recording of road traffic crash data since July 2017 to June 2018 in Iran.

**Participants and data generation method**

In this study, the participants were the people who played a key role in collecting and recording data about death and RTIS. They included the authorities of the Emergency organizations, police, Ministry of Health and Medical Education, faculty members and epidemiologic researchers in RTI prevention field, as well as executive staff and road users who were involved in collecting and recording data (n_15). The inclusion criteria were willing to participate in the study and having at least two years of work experiences or participating in decision-making or policy making in relation to the study aim.

Selection of participants in this study was primarily purposeful. In the first phase of participant selection and in order to achieve different opinions, maximum diversity sampling method was used among targeted participants. Participants' selection levels are shown in Table 1. The second phase was done based on theoretical sampling. Theoretical sampling is one of the important features of the grounded theory. Theoretical sampling is a tool for achieving theoretical saturation. When no new category of data is obtained and no new concept is added to the created categories, the theoretical saturation is created.^17^ In this study, information saturation was obtained with 15 interviews.

**Table 1:** Levels of participant selection in the study of the process of collecting and registering road traffic crash in Iran.

Number	Participants level	Stakeholders
7	Top level	Bosses and managers and vice-presidents of beneficiary organizations
3	Middle level	Faculty members and researchers
5	Operational levels	Victims, recorder staff at incident scene, hospital, police, forensic, emergency organization

**Data generation strategy**

The semi-structured face-to-face interview was used to generate data. The interview guide was designed based on the objectives of the study and was designed through three non-structured interviews, review of the studies and comments of the research group. The guide was designed to provide an understanding of the process of collecting and recording mortality data and traffic crash victims, factors affecting this process, action and interaction strategies, and outcomes of the process. Examples of questions include: What is your opinion about the location and structure of RTIs registration? What is your personal experience of collecting and recording road traffic crash information? How do you assess the status of collecting and recording road traffic crash in the country?

In order to conduct interviews with the participants, after telephone coordination, the researcher arrived at the appointed time and location determined by the participants. Then, they provided explanations about the study objectives and the confidentiality of the information, and the consent of the participants for the interview was received. Then, the interview was conducted by principle investigator (SSH) in Farsi. The content of the interview with the participants was recorded by two digital audio recorders, and during the interview, the note taking method was used for questioning in subsequent interviews. At the end of the interview, participants were requested to contact us in case of need for further information. The average interview time was 40 minutes. Audio files were transcribed immediately after the end of interviews, and then all handwritten texts were typed with Word Office 2010.

**Data analysis method**

Data analysis was performed after listening several times to audio file and re-matching of the transcribed content with the recorded voice. The Strauss and Corbin 2008^18^ method was used to analyze the data. In this method, data analysis is based on three stages including open coding for initial data analysis, axial coding for classification of the extracted content, and finally selective coding to explain the research theory. During open coding, the focus of the research group was on the construction of concepts. To this end, after repeated study of interviews, the concepts of data extraction were identified. In cases where the words or expressions were clear, the investigator used it as an invivo code. During the interviews and data analysis, the method of continuous comparison was used and the extracted codes were based on difference or similarity in the categories. Then the primary categories merged with each other using constant comparisons and formed new categories. In an axial coding analysis, the initial codes were compared to find the relationships between categories and subcategories, based on their similarity and differences. Then the groups having relations with each other were placed in a cluster. Finally, the main categories were formed on the basis of their conceptual relationship with each other. Concepts and categories formed on the basis of the Strauss-Corbin paradigm model, of which were found in the axis, casual, intervening categories, strategies, context, and eventually the consequences. 

In the grounded theory, the core variable is the idea and thought of the axis, the event or a phenomenon to which the flow of actions and reactions are leading in order to manage, control, or respond to it. The causative factors are the circumstances that affect the main cause and result in the occurrence or spread of the desired phenomenon.^19^

A structure or context is a set of specific features that refer to the phenomenon concerned and indicate a set of specific conditions in which action and interaction strategies take place. Intervening factors are the conditions that belong to the phenomenon and affect the action/interaction strategies. They facilitate or restrict strategies within a particular context. Strategies are targeted actions and responses that are used to control, manage, and deal with the phenomenon concerned. Consequences are the results of the strategies. Outcomes are not necessarily the things people intended to do. They may be real or implied or negatively affected, or may occur in the present or in the future.^16^

Finally, in the selective coding step, applying constant comparison and the relation between categories was helpful in choosing the core variable. After saturation of concepts, the relationship between concepts and the connection between each and the core variable was determined.^19,20^

**Trustworthiness**

To ensure data quality in grounded theory approach, two criteria including methodological stability, and quality and applicability were used based on Strauss and Corbin criteria.^20^ To ensure the stability of the methodology, the participants were identified and selected at different levels to collect information. Also, the consent form was obtained from interviewees. Moreover, theoretical saturation was performed and this issue was mentioned in the method section and the coding process was presented in the analysis section and the core category was extracted. Some of the participants were also provided the initial codes to ensure consistency of the findings with the participants' experiences.

In order to ensure the quality and application of the results, the axial categories and their relationship with other subcategories were extracted in the form of a conceptual model. In addition, all the categories were based on the experiences of the participants and relevant quotations were provided for the categories. In addition, the application of the study results in the discussion and conclusion section was presented. 

## Results

**Figure 1:**
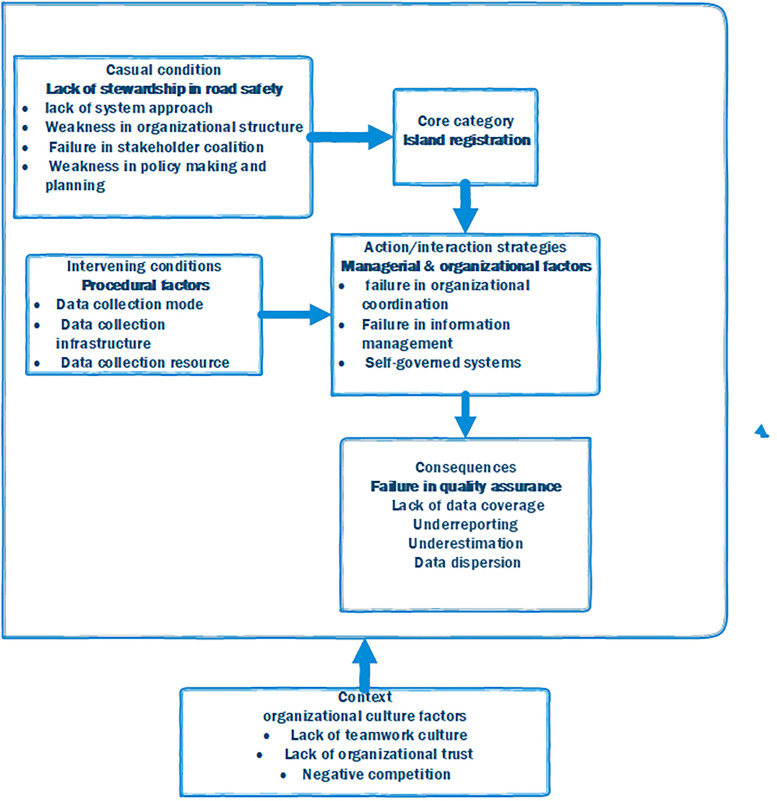
Conceptual model for road traffic data collection and registry in Iran.

According to the data analysis, based on grounded theory, a total of 5 categories and 17 subcategories were obtained from 800 primary codes. Based on the participant's viewpoint, separated registration was identified as the core variable. Other variables were categorized as casual factors, intervening, strategies, and outcomes (Figure 1).

**Core category**

To ensure the relationship between the core variable and other variables, the authors tried to examine the role of this variable in all categories and extracted codes. In this study, separated registration was identified as a variable that affects or takes effect from all codes and subcategories in the process of collecting and recording traffic crash information. In the process of collecting and recording RTIs data, separated registration and non-systematic data collection were reported as one of the main concerns of participants in this study. The point of interest in this category is that all organizations involved in road traffic incidents are registering relevant information, although this information may be incomplete or unnecessary. 

*... (P04) everyone works for himself on a separated paradigm and parallel to each other ..."*

**Casual factors**

According to the participants, the most important factor affecting separated registry is the lack of a road safety system in the country, and therefore, the functions of the lead agency are defective. Hence, in such a situation, a systematic perspective is not dominant. The existence of a custodian or a leading organization is very effective in creating a system approach, since all organizations in the field of emergencies are considered as one of the wholes, and the activities and roles of each one will affect each other, and all components of the system are in a process of interaction and affect each other. Therefore, due to the lack of this view, each stakeholder alone plays a role in the recording of traffic crash information. 

*(P02) ..." We do not have a definite manager for road traffic crash. The legislator gives the responsibility of the injured to the Ministry of Health, and Mortality to the Forensics Medicine Organization, and human errors to the police, these cause disruption..."*

In addition, due to the weakness of the structures in the RTIs beneficiary organization or lack of thinking about how to build a structure in an organization, several departments are obliged to carry out road traffic crash and injury prevention activities and programs, and because their duties are not separated, programs and activities are designed and implemented in a parallel and overlapping form. Based on the participants' point of view, RTIs stakeholders do not fully understand the goals and activities of each other. The tasks of the stakeholder organizations in the field of collecting information on RTIs are not clearly stated and, according to the participants, information is collected parallel and overlapped with relevant organizations. Furthermore, there are no specific rules for communication between stakeholders. In order to build stakeholder networks, it is important to build inter-organizational trust, and make clear rules for systematic communication. They believe that stakeholder communication is crucial to have a coherent and integrated registration system because information about traffic crash needs to be collected from a variety of sources, and without a stakeholder network this will not be possible. 

*(P07) ..." Everyone works his own way and we do not accept each other because we believe that our work is right and their work is wrong.*

In addition, according to the participants' views, one of the important functions of a RTIs lead agency is to determine the policies in this field. Unfortunately, due to the lack of clarity of overall policies and strategies on how to collect and record coherent road traffic crash information and the weakness of existing information management, each of the beneficiary organizations has their own indicators and methods for collecting information. Having multiple forms and trying to formulate and standardize each form of information collection in the beneficiary organizations will consume resources and time. By identifying general strategies in this regard, stakeholder organizations will become familiar with the role of the road safety and information system and how to use their information. RTI surveillance system will play an important role in this regard. 

*(P06)... We do not pay attention to resources and capabilities in designing the program. For this reason, we fail ... (P07) ... Unfortunately, things are done in a separated form..."*

**Structure and context**

In this study, the administrative and organizational structure and culture, affect the main phenomenon and therefore the action/interaction strategies of the organizations in the field of road safety. According to the participants, teamwork is a problematic issue in the stakeholder organizations in the majority of cases. They consider the need for a successful implementation of a coherent and integrated registration system to be teamwork and believing in it, and this should be done not only in the intra-organizational level, but also inter-agency and inter-organizations.

*(P10) ..." It's a fact that our weakness is teamwork. We have problems with every single thing that requires interworking and teamwork...*

Moreover, according to most participants, there is an atmosphere of distrust in the beneficiary organizations which is one of the situations and backgrounds in the administrative and organizational culture. This distrust, especially in the field of information, has become more significant and has affected the processes of collecting and recording information. According to the participants' opinion, organizations in this regard are pessimistic about how to collect and record each other's information. Participants also pointed to the existence of a competitive culture among stakeholders in this field. Accordingly, the organizations concerned are seeking to prove the accuracy and integrity of their activities and information, and they blame other stakeholders in this field for not having reliable information. The existence of negative competition between organizations has led each of them to seek to increase the quality and quantity of their information and avoid sharing information. 

*(P07) "... Everyone works his own way and does not accept each other because we believe methodologically that our work is right and their work is wrong*

**Intervening factors**

The analysis of the data obtained from the study demonstrated that the factors related to the methods of collecting and recording traffic crash data act as intervening factors on separated registration, inter-organizational mismatch and weakness of information quality. Data analysis showed that the status and methods of data collection are different among the beneficiary organizations. The goals of registration, the tools and types of variables in any organization can be a cause for inconsistency and lack of cooperation between organizations. In such a situation, due to the diversity of data collection methods and the type of recorded information, each organization, based on its organizational goals, collects and records information, which will affect the lack of inter-organizational coordination.

*"(P01)... The purpose of each organizations is different. The purpose of the registry office is different from the Ministry of Health...*

Participants thought that the weakness in existing infrastructure for collecting and recording information is an issue that challenges interpersonal communication and information management. Participants believed that the existence of technological infrastructure and applying modern technology in the country to upgrade existing systems and use of online registration methods can increase the inter-organizational relationship and, hence, enable inter-organizational collaboration and coordination. While in most parts of the country there is still no technical infrastructure for creating and improving online registration systems, this has made it difficult to establish an information link between organizations. Regarding the human resources required for registration, the participants also pointed to the importance of the existence of dedicated registrar users. While in many stakeholders, individuals who record data beside their routine tasks are used for the purpose of collecting and recording information. They have not been specifically trained to record traffic crash and injury data. They have not seen any special education. "

*(P09) ... the aim of HIS is financial management in the hospital, even where it records the cause of the injury. As a result, its goal is money, therefore the mechanism designed to collect its data is different... "*

According to most of the contributors and considering the analysis of existing data, another interventional factor can be the multiplicity of sources of road traffic crash and injury data collection and the diversity of the organization in this regard. Currently, most organizations, individually and locally, record information. Regarding the organizational goals of the stakeholders, the variables recorded by each of them are overlapping in many cases. Furthermore, the variables required by the surveillance system are scattered in different systems. In the opinion of the participants, the lack of proper understanding of the sources of information gathering among the stakeholders has caused the level of familiarity with each other's activities to be very small, and this will cause the beneficiary organizations to operate in this field alone with weak links to other stakeholders.

*"(P01) ... Now in our country, various organizations collect RTIs information, but there is no link between them and their variables do not match …"*

**Action/interaction strategies**

In this study, three sub-categories were identified as action/interaction strategies that were placed in a category called organizational factors. One of the most important strategies for separated registration is the lack of cooperation and coordination between organizations. In order to coordinate and interoperate in organizations, it is essential to change or match their systems and targets. Then in this case, organizations try to reduce their coordination and cooperation with other organization to resist the necessary changes. Moreover, they don’t feel any needs to cooperation and coordination due to the separation of the registration systems. 

*"(P05) ... We are not obligated to cooperate with other organizations in this field ... (P09) ... Follow the purpose of your organization, but we need to coordinate with each other when we want to make an injury surveillance, but this is very difficult in our country …"*

In addition, due to the separation of systems, the validity and reliability of the information recorded in each organization is not examined and compared. Organizations consider their registered data to be accurate considering predetermined goals. Most contributors believed that traffic crash and injury data were not efficient enough to make policy decision-making. RTI registry systems have weakness in recording indicators related to effective policy and decision making. In addition, in information management, joint programs between stakeholders are not implemented. Participants believe that organization collect parallel and overlapping information. Also, weakness in information management and lack of information links between stakeholders’ results in low-quality information.

*"(P05) ... There are about 10-15% of underreporting in traffic injury/fatality data. The quality of the registered information must also be improved... "*

In the opinion of the participants, management of road safety information is performed in each organization autonomously, and no attention is paid to the inter-organizational goals. All the steps for determining the structure, content, and the resources required for registration, are made separately. In this regard, resources may also be allocated to parallel activities repeatedly.

*"(P12) ... Policies for traffic crash are not specifically delegated. In my opinion, decisions must be made at one place …"*

**Consequences**

In this study, the consequences are lack of coordination, weakness in information management and the existence of autonomous systems in the field of collecting and recording information, which will lead to failure of quality assurance. The goal of quality assurance is to ensure that a particular process is achieved to the desired goal, so, monitoring and planning plays an effective role in in this regard. While the result of lack of coordination and autonomy of existing registry systems is to obtain information that is not well-suited for preventing and reducing the RTIs. In this study, the consequences of scattered and inconsistent registrations were identified, including the weakness of data coverage for RTIs and the data that was not suitable for planning and policy making. This weakness of coverage is also included in the coverage of the data recorded, and the coverage of the variables required for planning and policy. In the case of spatial coverage in some small villages and towns, registration systems in road traffic crash are inadequate, which has led to underreporting of mortality and injuries. In addition, the results from the analysis of the data showed that due to the weakness in this regard, priorities were not properly identified and there is underestimation of data to determine priorities and interventions.

*"(P09) ... We are having difficulty in assessing the quality of traffic crash data ... (P03) ... we have problems in tracking patient up to 30 days after the crash and we have no information about the disability ...*

Participants also acknowledged that one of the important implications for recording RTIs is the dispersion of data needed to plan traffic crash and injury. Their diversification and dispersion have made it difficult to create an integrated and consistent system for recording traffic crash data. 

*(P11) ... Now some data are in the Ministry of Health and Hospital, some data is in the Police Department and some is in the forensics…"*

## Discussion

To the best of our knowledge, this study as the first in its kind, explored to explain the model of collecting and recording RTIs information in Iran, which is based on qualitative study with grounded theory approach. The findings of this study have shown the relationship between various factors affecting the process of collecting and recording RTIs data in a conceptual model. This study showed that non-systematic and separated registration determined the status of collecting and recording RTIs system in the country. One of the most important factors affecting the data collection and recording system identified in this study was the lack of inter-institutional trust, lack of system approach, lack of lead agency, underreporting and underestimation.

The process of gathering and recording RTIs information in Iran is scattered, inter-organizational distrust is one of the factors influencing collecting and recording RTIs data procedure, which originates from organizational culture. Several studies have highlighted "trust" as a factor, which is the basis for creating interpersonal and organizational relationships and providing an interagency collaboration.^21-24^ According to the outcomes of this study, each organization, regarding its purpose, finds its method of collecting data and also the registered variables more credible and complete with respect to other organizations. Therefore, distrust between beneficiary organizations in collecting and recording at internal and external levels, is known as an underlying factor. Other studies also revealed that inter-institutional distrust leads to weakening the interpersonal and inter-organizational relationships, and ultimately leads to a lack of information exchange in organizations and a reduction in the quality of decisions made.^25,26^

In addition to confirming other studies on the impact of distrust on reducing interagency cooperation and information exchange,^25,26^ this study showed that the existence of inter-institutional distrust leads to a culture of falsification and inter-institutional blaming. RTI stakeholders are trying to blame the partner organizations for their lack of confidence in the information gathered and information collecting method of each other in this field. They also attribute any shortcomings or mistakes regarding the quality of information on RTIs in the country to defects of other systems. Inter-organizational trust plays a vital role in forming a surveillance system for RTIs victims. In this system, exchange and sharing of information between organizations is considered and regarding the importance of information as the source of power and assets of organizations, its exchange would be much harder with a culture of distrust and falsification. It seems that inter-institutional distrust can be overcome by increasing organizations recognition of each other's goals and performance as well as creating participatory programs among stakeholders in this field. Findings from other studies also confirm the impact of cognition and participation on increasing trust.^23,27,28^

This study showed that the absence of a legal guardian in RTIs is considered as a causal factor in non-systematic and separated registration. In most studies on RTIs information, lack of coherence and coordination of data generated in different sources is one of the challenges in this subject. In almost all studies, the need for a custodian and legal leader has been emphasized to support established programs and interventions.^9,13,29-31^

In the present study, it was found that due to various organizations at pre-incident and post-incident levels, there is a strong lack of integrated systems that can handle a large amount of information generated in these organizations. Other studies have also highlighted the importance of the role of lead agency in integrating and defining the purpose of data recording. They also stated that monitoring is one of the main features of the surveillance system, the most important components of which are the production of valid information for decision making, strategic planning, ensuring the official management of programs and interventions, effective connection between the stakeholders, preventing parallel works and monitoring.^32,33^ Regarding the components expressed in the monitoring, specifying a lead agency for the road safety would solve the issue of lack of coordination and separated registry of the traffic crash information spontaneously. However, the notable issue is to determine which of the existing organizations can be a legal agency for road safety of the country. Other causative factor identified in this study is the lack of a systematic approach to RTIs.

The importance of the existence of a system approach versus traditional approach to road safety has been mentioned in some studies.^34-38^ In this study, the importance of a systematic approach in this regard is that it makes both road users and beneficiary organizations responsible for improving safety and preventing RTIs. But according to this study, in Iran, the responsibility of the beneficiary organizations in this field is unknown and this issue has been affecting the formation of separated registrations and inconsistencies in traffic crashes. In collecting and recording RTIs information, the process of registering information in each of the RTIs organizations is different from other organizations, because there is no common responsibility between the beneficiary organizations in this area. Therefore, by choosing a system approach, the problem of creating a more coherent and complete registration, and consequently the design of joint interventions in this area can be improved.

The following factors were identified as interventional factors in forming separated registration: lack of coherent registry infrastructure, lack of clarity of recording information, and lack of required resources for registration in this study. In studies conducted in most low- and middle-income countries, it has been demonstrated that lack of infrastructure and sources of information is not specific to Iran alone, and exists in most countries.^30,39,40^ In Iran, according to the finding of this study, some organizations in this area do not have the technical infrastructure necessary for timely registration of information. In some organizations, including the pre-hospital system, there is no infrastructure required to record online information across the country. Therefore, systems collect and record information according to their limited resources. Elimination of technical and infrastructural weaknesses in collecting and recording information can prevent any disruption in coherent and integrated system for recording RTIs information.

In this study, lack of cooperation with stakeholders, challenge in the management of RTIs information and selection of self-centered systems are action strategies that organizations choose despite non-systematic and separated registration. However, due to the multidimensional nature of the traffic crash area and the numerous organizations involved in this area before, during and after the crash, and regarding the differences in the objectives of these organizations and the multiplicity of information, traffic information management have been encountered problems and in some cases there are reports less or more than reality.^38,41,42^ Following the diverse reports and statistics, the interventions have not been based on reality and have led policy makers and researchers to mislead policy interventions and safety promotion policies. 

According to our findings, beneficiary organizations, due to multiple registration systems are struggling to disagree with each other, because by accepting inter-organizational collaboration in creating a coherent register, there is a perception that changes to the system of collecting and recording each of the systems are created, so they resist the change and show this resistance as non-cooperation. Therefore, they prefer to have systems based on their limited goals, so they will not be required to cooperate with other interested organizations in the implementation of interventions and prevention programs. The existence of a coherent and integrated registration system and information sharing can enhance this collaboration.

The weakness in the quality assurance of information, as a consequence of separated registration, is stated in this study. In most of the studies conducted in this area, the underreporting and under-stimulation of RTIs data is mentioned.^43-46^ This study showed that separated registrations and inconsistencies resulted in poor quality of traffic crash information and, consequently, led to problems in estimation of injury burden in community groups, prediction of future programs and interventions, and future research in this field due to the dispersion of required data and lack of valid data. Moreover, design of effective interventions requires the identification of RTIs risk factors, and recognizing the risk factors of crash also requires accurate information on the situation of the injured at the time of the incident. 

In addition to data dispersion, some of the road traffic crashes risk factors are not collected or recorded due to lack of law or weakness in the implementation of existing laws. In Iran, due to the lack of rule law on maximum speed in urban roads, abuse of alcohol and drugs, the inability to use helmets and the lack of legislation on the use of child seats in the vehicle,^47^ information about the risks of the factors is not collected and not recorded. It is also important to collect RTIs location for better management, as other studies also indicated for the benefit of hot spot analysis for better management.^48,49^ It seems that registration of risk factors is not taken seriously by the relevant organizations due to RTI separated registration systems. Therefore, data are to be collected in a targeted manner in order to use the information and to apply this information.

**Strengths and limitations**

One of the strengths of this study is using the various views of the participants who are stakeholders in the process of collecting and recording RTIs data, who were experts in this field. Due to the use of the grounded approach, all of the concepts and theories created in this study were saturated, and all views and experiences of individuals in this area were examined. One of the limitations of the study was gathering data from different and diverse organizations involved in RTIs, and it made collecting information and creating inter-organizational coordination difficult.

## Conclusion

This study describes important theory of the status of road traffic injury registration as separated and non-systematic. In the conceptual model of the factors, the strategies have been shown. The findings of this study will help policymakers to better understand the situation of collecting and recording road traffic injury information. It seems to be possible to enhance the process of collecting and recording road traffic injury information by increasing inter-organizational awareness and formulating joint and distinct responsibilities among beneficiary organizations and designing programs with benefits and rewards for stakeholders.

**Abbreviations**

SSH: Sakineh Sharifiyan, HSB: Homayou Sadeghi-Bazargani, RM: Reza Mohammadi, RKHJ: Reza Khani-Jazani, RTI: Road traffic injury, DKZ: Davoud Khorasani-Zavareh

**Declarations**

**Ethics approval and consent to participate**

This study is part of PhD thesis, with the IR. SBMU.RETECH.REC.1396.206 ethics code in July 2017 in Shahid Beheshti University of Medical Sciences. In each interview, the purpose of the study was explained to the participants. Participants' names were confidential and numerical codes were used instead. In all stages of the interview, the independence of the participant for taking part, and the exclusion of research was maintained.

**Acknowledgement**

This paper was part of a PhD thesis in School of Public Health and Safety, Shahid Beheshti University of Medical Sciences (SBMU).
